# Effectiveness and safety of secukinumab updosing in patients with moderate to severe plaque psoriasis: data from the PURE registry

**DOI:** 10.1007/s00403-024-03122-w

**Published:** 2024-06-08

**Authors:** Kim A. Papp, Melinda Gooderham, Charles Lynde, Danielle Brassard, Faisal Al-Mohammedi, Vimal H. Prajapati, Isabelle Delorme, Lorne Albrecht, Richard Haydey, Maryam Shayesteh Alam, Jennifer Beecker, Sanjay Siddha, Marie Maguin, Mahmoud S. Farag, Antonio Vieira, Lenka Rihakova, Richard G. Langley

**Affiliations:** 1 Alliance Clinical Trials and Probity Medical Research, Waterloo, ON Canada; 2 The University of Toronto, Toronto, ON Canada; 3 SKiN Center for Dermatology, Queen’s University and Probity Medical Research, Peterborough, ON Canada; 4 Lynde Institute for Dermatology, University of Toronto and Probity Medical Research, Markham, ON Canada; 5 Clinique D, Laval, QC Canada; 6 Dermcare Clinic and, University of Toronto, Mississauga, ON Canada; 7 Division of Community Pediatrics and Pediatric Rheumatology, Department of Pediatrics, University of Calgary, Calgary, AB Canada; 8 Division of Dermatology, Department of Medicine, University of Calgary, Calgary, AB Canada; 9 Dermatology Research Institute and Probity Medical Research, Calgary, AB Canada; 10 Skin Health & Wellness Centre, Calgary, AB Canada; 11 Dr. Isabelle Delorme Inc, Drummondville, QC Canada; 12 Enverus Medical Research, University of British Columbia and Probity Medical Research, Surrey, BC Canada; 13 Winnipeg Clinic, Winnipeg, MB Canada; 14 Simcoderm Medical and Surgical Dermatology Centre and Probity Medical Research, Barrie, ON Canada; 15 University of Ottawa, Ottawa, ON Canada; 16 Division of Dermatology, The Ottawa Hospital, Ottawa Hospital Research Institute and Probity Medical Research, Ottawa, ON Canada; 17 Probity Medical Research, Division of Dermatology, University Health Network Hospitals, Toronto, ON Canada; 18 Novartis Canada Pharmaceutical Inc, Montreal, Canada; 19 Division of Clinical Dermatology & Cutaneous Science, Department of Medicine, Halifax, Canada

**Keywords:** Investigator’s Global Assessment, Plaque psoriasis, Psoriasis Area and Severity Index, Secukinumab, Updosing

## Abstract

Secukinumab is a fully human IgG1 antibody that selectively binds to and neutralizes the proinflammatory cytokine interleukin-17A. Secukinumab is an effective and well-tolerated treatment for plaque psoriasis. There is a limited real-word evidence for dose optimisation of secukinumab based on clinical response. PURE is a multi-national, prospective, observational study in patients with moderate to severe chronic plaque psoriasis in Canada and Latin America, assessing the real-world safety and effectiveness of secukinumab and other indicated therapies. The aim of the current snapshot analysis was to evaluate the effectiveness and safety of on-label dose and updosed secukinumab in patients with plaque psoriasis enrolled in the PURE study. At the time of analysis, 676 patients received secukinumab, of which 84.6% (*n* = 572) remained on the on-label dose, while 15.4% (*n* = 104) were updosed. With on-label secukinumab, the absolute Psoriasis Area and Severity Index (PASI) score was reduced from 13.6 at baseline to 1.2 over 36 months, with treatment persistence of 73% at 40 months. At Month 36, 73.2% of the patients receiving on-label secukinumab achieved Investigator’s Global Assessment (IGA) 0/1. With updosed secukinumab (300 mg every 2 weeks, 300 mg every 3 weeks, 450 mg every 4 weeks, or 450 mg every 3 weeks), 57.9% of the patients showed improvement in the absolute PASI score at the first visit after updosing, with treatment persistence of 50% at 12 months after updosing. At Month 15, 40% of patients receiving updosed secukinumab achieved IGA 0/1. Patients with previous biologic exposure (odds ratio [OR]: 3.25; 95% confidence interval [CI]: 2.03, 5.18, *p* < 0.0001) were more likely to be updosed while those with a body weight < 90 kg (OR: 0.49; 95% CI [0.31, 0.77], *p* = 0.0019) were less likely to be updosed. Previous biologic exposure (HR [hazard ratio]: 1.47; 95% CI [1.24, 1.75], *p* < 0.0001) and current biologic exposure (secukinumab vs. other indicated therapies: HR 0.57; 95% CI [0.43, 0.75], *p* = 0.0001) were significantly associated with time to secukinumab updosing. No new or unexpected safety signals were observed with updosed secukinumab. Secukinumab updosing was efficacious and well-tolerated in patients with psoriasis who failed to respond to the approved on-label regimen, suggesting that updosing may be a useful therapeutic option for approved dose non-responders.

## Introduction

Psoriasis is a chronic, immune-mediated, inflammatory condition with manifestations that can involve the skin, nails, joints and other organ systems. With a worldwide prevalence ranging from 0.6 to 7% of the population, psoriasis affects up to 2–5% of adults in Western countries [1] and is associated with a high degree of morbidity. Biologics are primarily indicated for patients who are candidates for systemic therapy or phototherapy. The four main classes of biologics approved for the treatment of moderate to severe plaque psoriasis include the tumour necrosis factor alpha (TNF-α) inhibitors, interleukin (IL)-12/23 inhibitors, and IL-17 inhibitors [2].

Secukinumab, a fully human monoclonal antibody, selectively neutralises IL-17A, a cornerstone cytokine involved in the pathophysiology of psoriasis. The efficacy and safety of secukinumab in patients with moderate to severe chronic plaque psoriasis has been well-established in pivotal phase 3 clinical trials. These studies have demonstrated long‐lasting effects of secukinumab in treating the complete spectrum of psoriasis (including the scalp, nails, palms and soles), psoriatic arthritis, and ankylosing spondylitis [3–6]. Some patients may benefit from dose optimisation of the dosing regimen based on their individual responses. In recent years, considerable evidence on the effectiveness and safety of secukinumab has emerged from real-world experience through pharmaceutical company–sponsored or independent registries and post-marketing phase 4 studies [7–9]. Nonetheless, there is limited data examining the potential role of updosing secukinumab to optimise the treatment regimen for the desired efficacy outcomes of clear to almost clear skin.

PURE is a multinational, prospective, observational cohort study in patients with moderate to severe chronic plaque psoriasis in Canada and Latin America (Argentina, Brazil, Costa Rica, Guatemala, Mexico, Panama and Dominican Republic), assessing the real-world safety and effectiveness of secukinumab and other indicated therapies (NCT02786186). This report from the PURE registry provides an insight into the clinical outcomes and treatment persistence of secukinumab in patients who were updosed compared with those who were not until 05 June 2019.

## Materials and methods

### Study design

The PURE registry, an ongoing study, aimed to enroll approximately 2,500 adult patients from 81 community and hospital specialty sites across Canada and Latin America. The study comprises two different patient cohorts, 1250 patients each. At baseline, patients received treatment with either secukinumab (Cohort 1) or other indicated therapies (Cohort 2). Due to the longitudinal and observational nature of the study, treatment switch can occur. The decision to treat in any of these cohorts was reached prior to and independently of recruitment in the study. Symptomatic patients (≥ 18 years) diagnosed with moderate to severe chronic plaque psoriasis by a specialist were included in the study. All treatment decisions, including the need for updosing, were based on the clinical judgment of the treating physician. The study includes a 5-year follow-up at completion, with recommended assessments at enrolment, 3 and 6 months and every 6 months thereafter. At any time, patients were given the option to withdraw consent and discontinue the study. The treating physician could also decide to withdraw a patient from the study on their discretion at any given point. The study enrolment was completed on 31 December 2020. The detailed study design including eligibility criteria of the PURE registry have been previously described [10].

### Outcome measures

Baseline demographics and disease characteristics were described for both the on-label and updosed secukinumab-treated patient populations. The treatment outcomes were evaluated for all patients at baseline, month 3, month 6 and every 6 months afterwards as per the study design. For the updosed secukinumab-treated patient population, treatment outcomes were evaluated before updosing and post-updosing (month 3 and every 6 months afterwards). Effectiveness assessment included Psoriasis Area and Severity Index (PASI) and Investigator’s Global Assessment (IGA) scores. Treatment persistence over time was also assessed. Safety was evaluated by measuring the number of total adverse events (AEs), total serious AEs (SAEs) and total severe AEs.

### Statistical analysis

Since dose optimization is facilitated in Canada, all updosed patients in this analysis were from Canada. A multiple logistic regression was performed for patients who were updosed by employing baseline demographic and clinical characteristics including age, sex, biologic exposure, weight (≤ 90 kg vs. >90 kg) as well as PASI, IGA, and Dermatology Life Quality Index (DLQI) scores. These variables were also used to analyse the time to secukinumab updosing using a Cox proportional hazard analysis.

The modified intent-to-treat population (mITT) includes all enrolled participants with an assigned cohort at baseline that had at least one post-baseline visit or electronic patient-reported outcome (ePRO) submission and/or a post-baseline AE evaluation. All patients who started on secukinumab at any time point in this study were pooled regardless of their cohort at enrollment. Clinical characteristics of the patients were compared between treatment cohorts using the independent-samples t-test and Chi-square test for continuous and categorical variables, respectively. Data are reported as mean ± standard deviation (SD) or in the form of percentages. For both treatment cohorts, AEs and SAEs were assessed using the total number of events, the number of patients and percentage of patients who experienced at least one event within individual system organ class and within individual preferred term described in Medical Dictionary for Regulatory Activities (MedDRA).

## Results

### Baseline assessments

As of 05 June 2019, 1,773 patients (Cohort 1/Cohort 2: 720/1053) from Canada and Latin America were enrolled in the PURE registry, of whom 1,512 were in the mITT population (Cohort1/Cohort2: 635/877). Among the mITT population, 676 patients (Cohort1/Cohort2: 609/67) received secukinumab, where 84.6% (*n* = 572; Cohort 1/Cohort 2: 517/55) remained on the on-label dose and 15.4% (*n* = 104; Cohort 1/Cohort 2: 92/12) were updosed. During the observation period, only five (4.8%) of the total updosed patients discontinued the secukinumab treatment.

At baseline, the mean age of the updosed patients was similar to those on the on-label dose, but weight and proportion of patients with previous biologic exposure were both higher in updosed patients (mean [SD] weight, kg: 100.4 [26.1] vs. 90.9 [22.8]; previous biologic exposure: 64.4% vs. 36.0%; Table 1). Most of the updosed patients were administered a secukinumab regimen of 300 mg every 2 weeks (Q2W) (Table 2).

**Table 1:** Baseline demographics and disease characteristics for secukinumab-treated patients who received on-label dose and those who were updosed

Characteristics	On-label dose (*N* = 572)	Updosed (*N* = 104)
**Age (years), mean (SD)**	49.9 (13.9)	49.5 (13.1)
**Sex, male, n (%)**	353 (61.7)	59 (56.7)
**Weight (kg), mean (SD)**	90.9 (22.8)	100.4 (26.1)
**Weight group, n (%)**		
**n (%)**	538 (94.1)	100 (96.2)
< 90 kg	293 (51.2)	35 (33.7)
≥ 90 kg	245 (42.8)	65 (62.5)
**Duration of psoriasis (years), mean (SD)**	17.1 (13.2)	20.1 (13.0)
PASI, * mean (SD)	13.9 (8.9)	12.3 (9.1)
PASI, * median (range)	12.0 (0.0, 70.8)	10.1 (1.8, 52.2)
**IGA score, n (%)**		
0	1 (0.2)	0 (0.0)
1	0 (0.0)	0 (0.0)
2	8 (1.4)	3 (3.3)
3	389 (68.2)	69 (75.0)
4	169 (29.6)	20 (21.7)
**DLQI, mean (SD)**	13.6 (7.42)	13.4 (7.16)
**Patients with prior biologic exposure at study enrolment, n (%)**	206 (36.0)	67 (64.4)
**Number of prior biologics prior to study enrolment, n (%)**		
1	121 (21.2)	25 (24.0)
2	55 (9.6)	23 (22.1)
3	20 (3.5)	13 (12.5)
4 or more	10 (1.7)	6 (5.8)

*Baseline PASI indicates disease severity at registry entry.

DLQI, Dermatology Life Quality Index; N, total number of patients; n, number of patients;

PASI, Psoriasis Area and Severity Index; SD, standard deviation.

**Table 2:** Distribution of different dosing regimens for secukinumab-treated patients who were updosed

Updosing strata	*n* (%)
N	104
300 mg Q2W	61 (58.7)
300 mg Q3W	29 (27.9)
450 mg Q4W	12 (11.5)
450 mg Q3W	2 (1.9)

N, total number of patients; n, number of patients; Q2W, every 2 weeks; Q3W, every 3 weeks; Q4W, every 4 weeks

### Treatment persistence

**Fig. 1:**
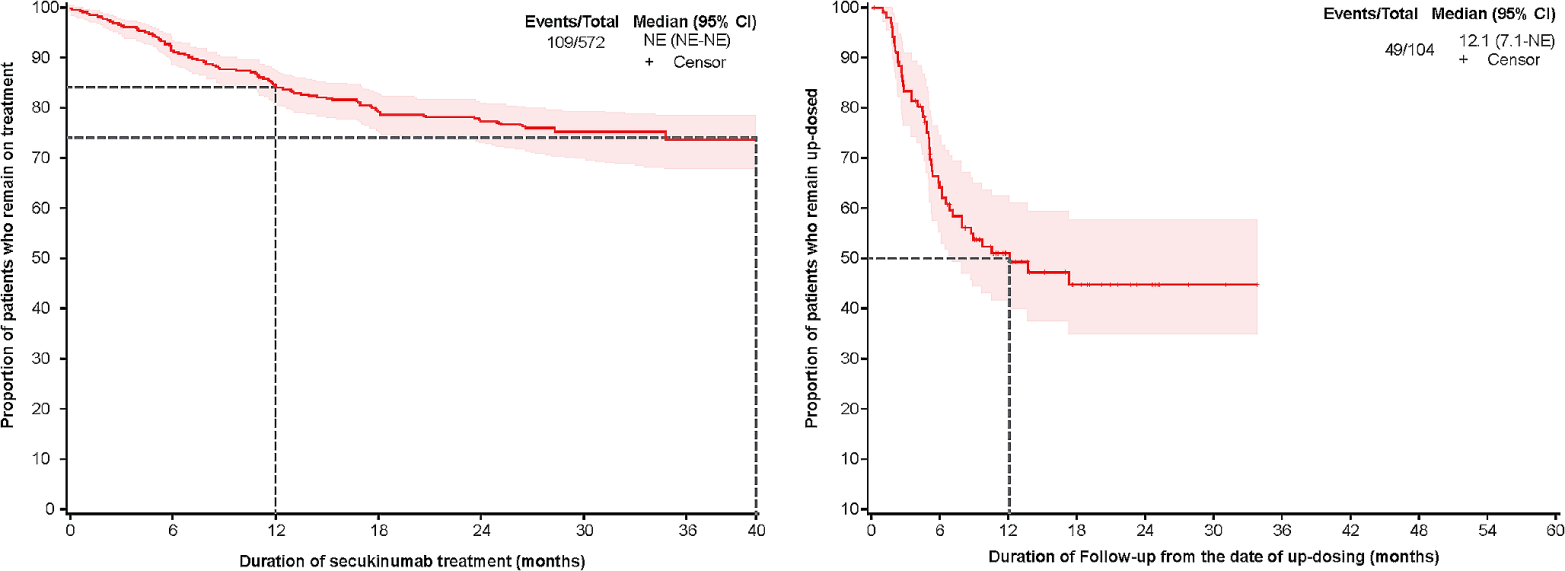
Treatment persistence in secukinumab-treated patients (**A**) who were on the on-label dose (**B**) who were updosed. CI, confidence interval; n, number of patients; NE, non-evaluable

The treatment persistence for patients who were on the on-label dose of secukinumab was high (~ 84% at 12 months and 73% at 40 months; Fig. 1A). The median time patients remained on updosed secukinumab was shorter at 12.1 months (Fig. 1B).

### Disease severity of patients on the on-label dose at enrolment and up to 36 months of follow-up

**Fig. 2:**
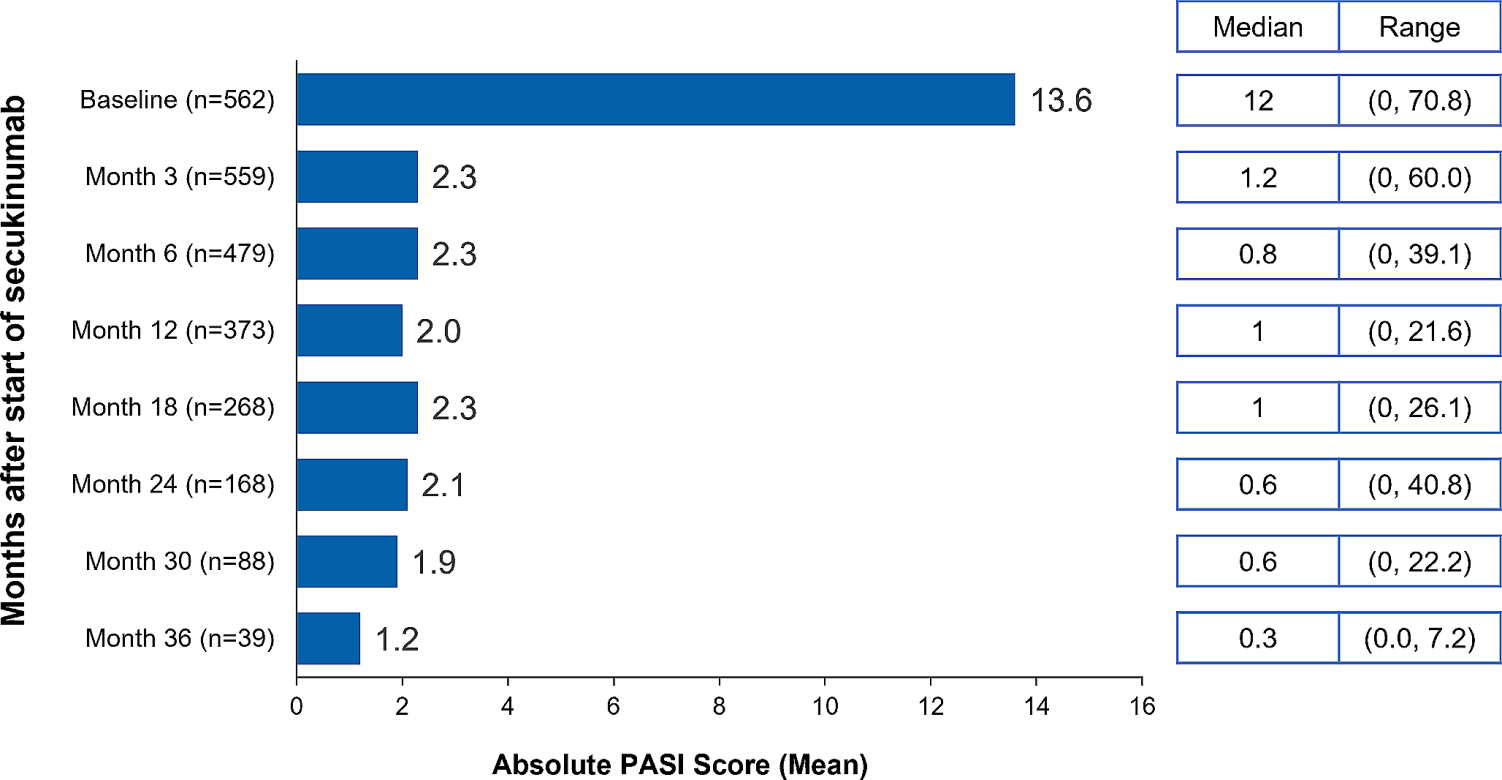
Mean PASI scores over time in secukinumab-treated patients on the on-label dose. n, number of patients; PASI, Psoriasis Area and Severity Index. Data represented for patients with a PASI score available at a particular visit after the start of secukinumab

**Fig. 3:**
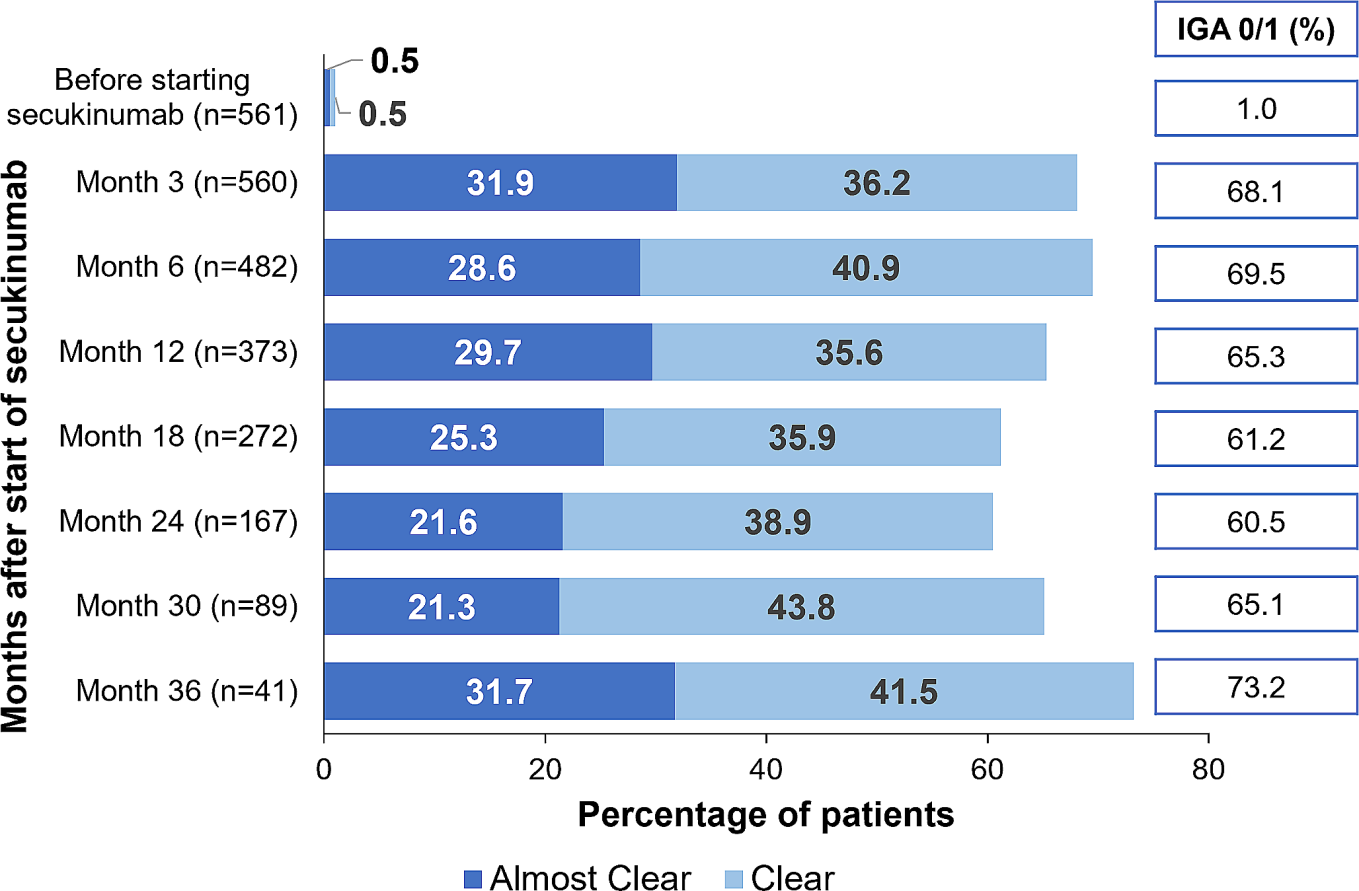
Percentage of patient achieving IGA 0/1 over time in secukinumab-treated patients on the on-label dose. n, number of patients; IGA, Investigator’s Global Assessment. Data represented for patients with an IGA score available at a particular visit after the start of secukinumab

Overall, patients who remained on the on-label dose maintained low mean absolute PASI scores over time; those with available 36-month follow-up (*n* = 39) had a mean absolute PASI (SD) of 1.2 (1.8) (Fig. 2). The median absolute PASI score remained stable over time during the follow-up period up to 36 months, with values below 1 (range: 0.0-7.2). Overall, at 36 months, 73.2% of the patients achieved clear or almost clear skin (IGA 0/1) and 41.5% of the patients achieved clear skin (IGA 0) (Fig. 3).

### Time to updosing

**Fig. 4:**
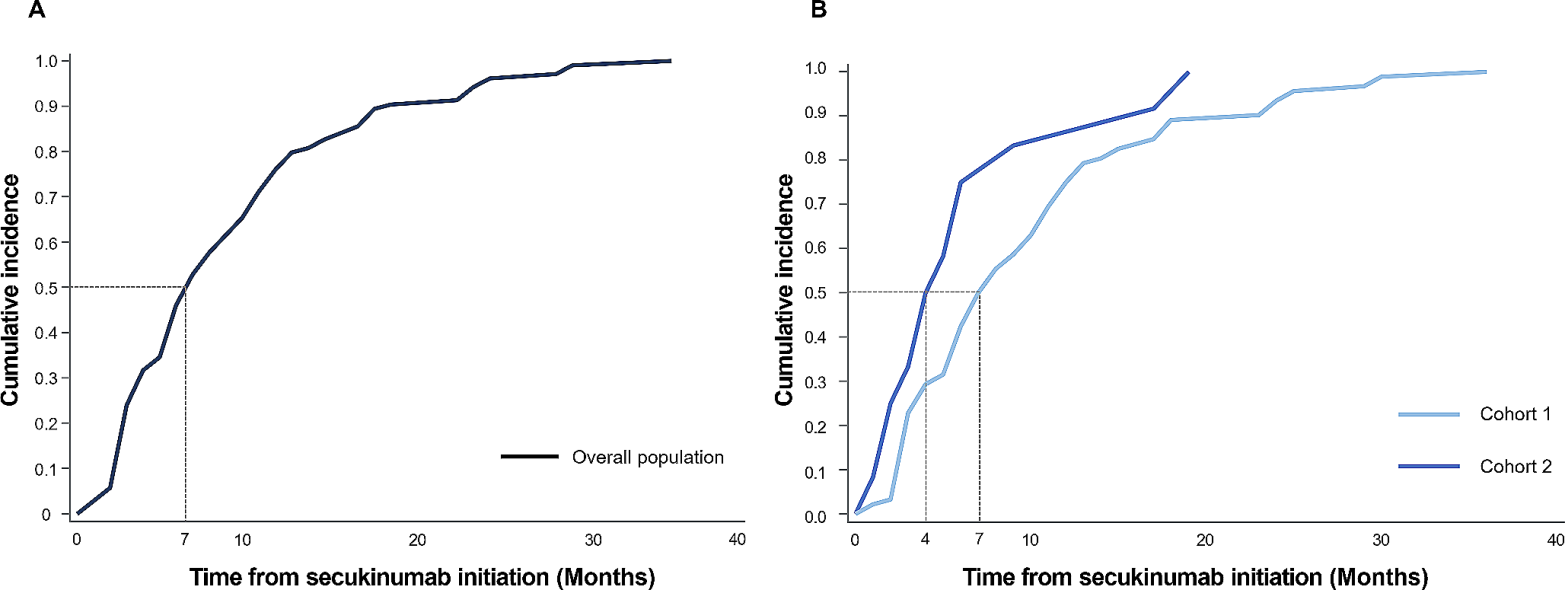
Time to updosing (**A**) Cumulative incidence of updosing of secukinumab in overall population (**B**) Cumulative incidence of updosing of secukinumab by cohort

The time to updosing after updosed secukinumab initiation in the 104 patients is shown in Fig. 4. The overall mean time was 7 months (range: 1–36 months) (Fig. 4A). Both mean [SD] (9.8 [7.5] vs. 6.5 [5.8] months) and median times (7 [range 1–36] vs. 4 [1–19] months) to updose were slightly longer in patients from Cohort 1 than from Cohort 2 after secukinumab initiation (Fig. 4B).

### Disease severity of patients who were updosed at enrolment and up to 30 months of follow-up

#### PASI score

Patient-level data analysis was performed for patients whose PASI scores were available after updosing. A total of 95 of the104 updosed patients had available PASI score measures. Among these, 89 patients who experienced a change in the PASI score after updosing of secukinumab, 55 of 95 (57.9%) patients benefited from updosing (as defined by improvement of their absolute PASI data after updosing) and experienced a further mean decrease of disease activity by 3.3 points for the absolute PASI score at first visit after updosing. The remaining 34 of 95 (35.8%) patients experienced further disease worsening, with a mean increase of 3.3 points on average for the absolute PASI score at first visit after updosing. Six patients had no change in PASI after updosing and data for nine patients were missing. In patients who were updosed, the mean PASI (SD) score remained stable from 5.0 (3.8; *n* = 101) at the visit before updosing to 3.8 (4.0; *n* = 20) at 15 months after updosing. However, of note, the maximum recorded PASI score in updosed patients decreased from 17.0 at the visit before updosing to 9.6 at 20 months after updosing.

#### IGA score

**Fig. 5:**
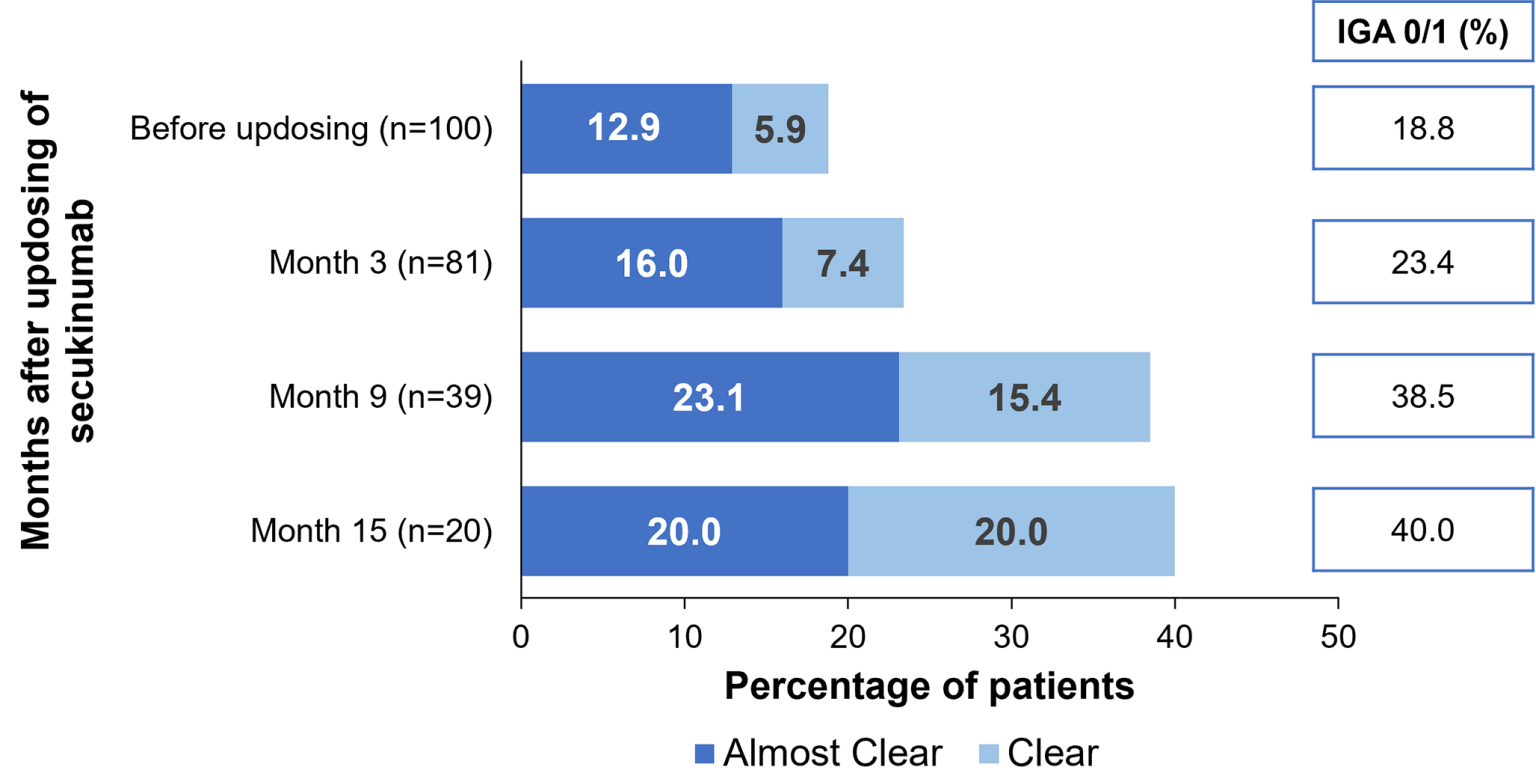
Effect of secukinumab updosing on proportion of patients achieving IGA 0/1. n, number of patients; IGA, Investigator’s Global Assessment. Data represented for patients with an IGA score available at a particular visit after secukinumab updosing

The mean change in IGA score from the visit before updosing in patients who were updosed remained stable (− 0.9 [1.0]; *n* = 100) to (− 1.6 [1.1]; *n* = 20) at 15 months after updosing. Moreover, 40% of the patients achieved IGA 0/1 and 20% of the patients achieved IGA 0 at 15 months after updosing, respectively (Fig. 5).

### Factors associated with probability of updosing

**Fig. 6:**
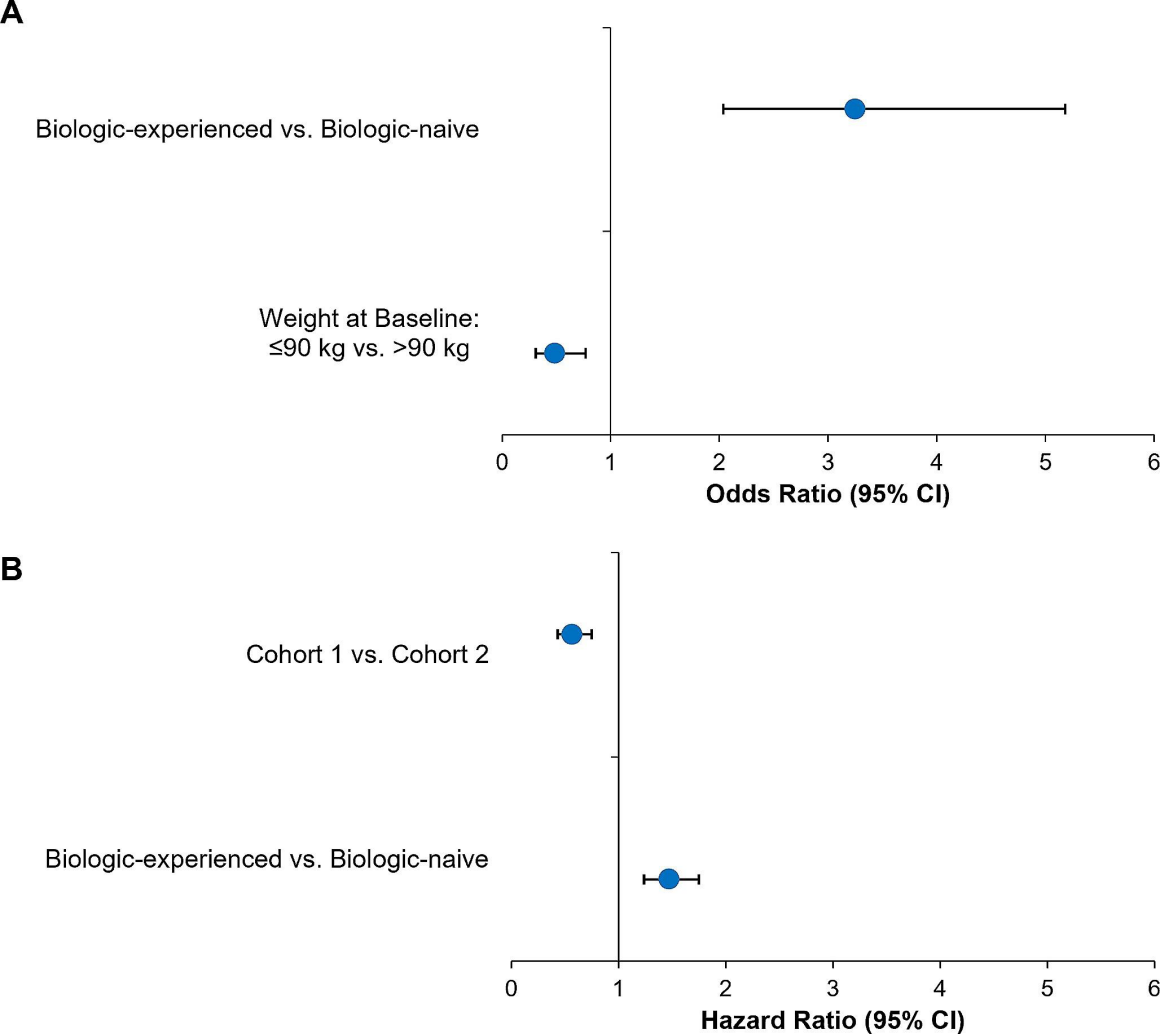
Multiple regression analysis **A**) Factors associated with probability of updosing; **B**) Factors associated with the time to secukinumab updosing. CI, confidence interval

Multiple logistic regression analysis, which included baseline demographics, clinical characteristics, and treatment parameters as covariates, revealed that prior biologic exposure and higher weight at baseline were significantly associated with updosing. Patients who were biologic-experienced were more than three times more likely to be updosed with secukinumab (odds ratio [OR]: 3.25, 95% confidence interval [CI] [2.03, 5.18]; *p* < 0.0001) than patients who were biologic-naive. Patients who had body weight ≤ 90 kg were less than half as likely to be updosed with secukinumab than patients with baseline body weight > 90 kg (OR: 0.49, 95% CI: [0.31, 0.77], *p* = 0.0019; Fig. 6A).

### Factors associated with the time to secukinumab updosing

Cox proportional hazard analysis was conducted to examine factors influencing the time to secukinumab updosing. Previous biologic exposure (hazard ratio [HR]: 1.47, 95% CI [1.24, 1.75]; *p* < 0.0001) and current biologic exposure (secukinumab [cohort 1] vs. other indicated therapies [cohort 2]: HR 0.57, 95% CI: [0.43, 0.75]; *p* = 0.0001) were significantly associated with time to secukinumab updosing (Fig. 6B). There was a small but statistically significant negative relation between baseline PASI (estimate − 0.015, *p* = 0.0129) and DLQI (estimate − 0.0117, *p* = 0.0499) and the probability of updosing was of marginal significance.

### Safety

Overall, secukinumab was well-tolerated and the safety profiles were comparable before and after updosing. There were no AEs leading to hospitalization and/or prolongation of existing hospitalization, and patients did not experience any SAEs after updosing with secukinumab (Table 3). One patient died after updosing due to subarachnoid hemorrhage; this AE was not suspected to be related to the study drug.

**Table 3:** Safety profile of secukinumab in patients before and after updosing

Characteristics, * *n* (%)	Event starting prior to updosing (*N* = 104)	Event starting after updosing (*N* = 104)
**Patients with any AEs**	70 (67.3)	55 (52.9%)
Any AEs related to study treatment	25 (24.0)	23 (22.1)
Any AEs leading to treatment discontinuation	13 (12.5)	14 (13.5)
Any AEs leading to hospitalisation/prolongation of existing hospitalisation	1 (1.0)	0 (0.0)
**SAEs**		
Patients with any SAEs	4 (3.8)	1 (1.0)
Any SAEs related to study treatment	1 (1.0)	0 (0.0)
Any SAEs leading to treatment discontinuation	0 (0.0)	1 (1.0)
Any SAEs leading to hospitalisation/prolongation of existing hospitalisation	2 (1.9)	0 (0.0)
Any SAEs leading to death	0 (0.0)	1 (1.0)
**Patients with any severe AEs**	4 (3.8)	3 (2.9)

*Patients who experienced the same event multiple times were only counted once for the corresponding system organ class and preferred term.

AE, adverse event; N, total number of patients; n, number of patients; SAE, serious AE.

## Discussion

The study results demonstrate real-world evidence of the effectiveness and safety of secukinumab updosing in patients with moderate to severe chronic plaque psoriasis in Canada. We sought to determine whether patients with an inadequate response to on-label secukinumab could further improve responses when treated with updosed secukinumab (300 mg Q2W, 300 mg every 3 weeks [Q3W], 450 mg every 4 weeks [Q4W], or 450 mg Q3W; based on clinical judgment of the treating physician) compared to when these patients were treated with on-label secukinumab (300 mg Q4W). The 84.6% of secukinumab-treated patients who continued with the on-label dose showed improvements in absolute PASI and IGA scores over a period of 36 months. Among secukinumab-treated patients who were updosed, 57.9% showed improvement in absolute PASI scores over 15 months. Half of the updosed secukinumab-treated patients remained on treatment 12.1 months after being updosed. Interestingly, the baseline PASI for patients who were updosed was slightly lower than patients receiving on-label dose. A previous study corroborates with this observation, where patients receiving high dose of secukinumab (300 mg Q2W) had low mean ± SD baseline PASI compared to patients receiving the on-label dose [11]. In this study, with over 36 months of treatment with the on-label dose, the mean baseline PASI value was reduced from 13.6 to 1.2 and treatment persistence was 73% at Month 40. These findings are similar to that observed in a previous real-world study, SERENA, where administration of secukinumab 300 mg Q4W for 36 months reduced a higher baseline PASI from 21.0 to 1.9 with a treatment persistence of 60.5% [7]. The mean baseline PASI in this study was also lower than the two pivotal phase 3 randomised controlled trials (RCTs) of secukinumab (ERASURE: 22.5; FIXTURE: 23.9) [3]. The reduction in the baseline PASI score at 36 months as reported is in line with that reported in the 3-year data of the ERASURE-FIXTURE extension study [12]. The study also demonstrated that with the on-label dose, consistent improvement in IGA 0/1 was observed from month 3 onwards. This finding is similar to that in the CLEAR study where rapid improvement in IGA 0/1 was observed at around month 3 with the on-label dose [13].

The ONDA study reported that, the efficacy of several biologics such as etanercept, adalimumab, infliximab and ustekinumab improved with dose adjustments in patients with psoriasis and psoriatic arthritis [14]. Studies conducted with SEC for updosing are generally small in scale, involving a limited number of patients. Nonetheless, these studies demonstrated a potential for improved outcomes with secukinumab updosing. The previous real-world studies conducted in Canada demonstrated that 40–46% of patients achieved IGA 0/1 with updosing of secukinumab for ~ 3 months [15, 16]. A retrospective chart review demonstrated that secukinumab updosing (300 mg Q3W, 300 mg Q2W, or 450 mg once weekly [QW]) led to improved clinical response with minimal AEs in 14 patients with moderate to severe plaque psoriasis [16]. The GAIN study also revealed that secukinumab 300 mg Q2W could be beneficial in patients with moderate to severe psoriasis with suboptimal response to 300 mg Q4W (IGA 0/1: 73.0% vs. 64.1%, *p* < 0.05) [17]. These findings indicate that updosing could be beneficial for patients who demonstrated inadequate treatment response to the approved on-label dose. In this study, 57.9% of the patients who received updosed secukinumab showed an improvement in PASI score with a treatment persistence of 50% at 12.1 months. Moreover, at 15 months, 40% of the patients also achieved IGA 0/1 with updosed secukinumab. Of note, 35.8% of the patients showed further disease worsening (measured by PASI scores) despite updosing of secukinumab. which was also observed in previous study [16].

Compared with the patients who remained on the on-label dosing, updosed patients were slightly heavier and had nearly double the exposure to prior biologics. Moreover, it is shown that higher body weight (≥ 90 kg) is associated with decrease in the mean concentration of secukinumab, impacting the treatment response to secukinumab [18]. Since weight and previous biologic exposure are known to affect the treatment response [19–21], these characteristics in updosed patients may explain the inadequate response to the on-label dose. Nonetheless, the updosing improved the response rates in this harder-to-treat population. These findings are in line with previous studies where updosing of secukinumab improved the therapeutic response in heavier patients with prior biologic exposure [22–24]. This study also conducted multivariate analyses to identify the factors that could predict the need for secukinumab updosing. The results further confirm previous findings that heavier body weight ≥90 kg and biologic-experience were independent factors significantly associated with updosing [19–21]. Patients in Cohort 1 were less likely to be updosed as they were comparatively lighter and had less biologic exposure compared to those in Cohort 2. The OPTIMISE study reported that heavy patients (≥ 90 kg) who did not achieve a PASI 90 response at Week 24 with secukinumab 300 mg Q4W may experience benefit with secukinumab 300 mg Q2W [25].

Secukinumab updosing was well-tolerated in patients with moderate to severe chronic plaque psoriasis. The safety findings of this study are in line with those reported in secukinumab phase 3 RCTs [6–9, 12] and there were no new or unexpected AEs. Moreover, these safety finding are consistent with previous studies evaluating the safety profile of higher doses of secukinumab [17, 25].

In August 2022, the Canadian product monograph of secukinumab was revised. It now provides an option for dose optimisation from 300 mg Q4W to 300 mg Q2W in adult patients with plaque psoriasis with a body weight ≥ 90 kg [26]. We would like to highlight that our results in patients with plaque psoriasis from Canada, which were collected approximately 3 years prior to the aforementioned date for the product monograph revision, is aligned with the dose adjustments suggested by the Canadian product monograph.

A major limitation of the study is the low number of patients who were updosed. As an interim analysis, the number of patients with evaluable data at the time of data cut-off was low. In addition, we report pooled effectiveness and safety data of the updosed secukinumab whereas the effectiveness and safety profile for each individual updosing regimen (i.e., 300 mg Q2W and Q3W; 450 mg Q3W and Q4W) is not available. However, as the PURE registry is still ongoing, further data will be collected to assess effectiveness and safety profile of updosed secukinumab.

## Conclusion

This analysis from the PURE registry provides evidence that updosing of secukinumab can be beneficial and well tolerated in patients with moderate to severe chronic plaque psoriasis who do not respond adequately to the approved on-label dose. No safety concerns were identified during this analysis.

## Data Availability

The datasets generated and/or analyzed during the current study are not publicly available. Novartis is committed to sharing with qualified external researchers access to patient-level data and supporting clinical documents from eligible studies. These requests are reviewed and approved on the basis of scientific merit. All data provided are de-identified/anonymized to respect the privacy of patients who have participated in the trial in line with applicable laws and regulations. The data may be requested from the corresponding author of the manuscript.
